# Surgical management of growing skull fractures: How I do it

**DOI:** 10.1007/s00701-025-06646-w

**Published:** 2025-08-29

**Authors:** Manina M. Etter, Tamara Vernik, Maria Licci, Raphael Guzman, Jehuda Soleman, Ladina Greuter

**Affiliations:** 1 Department of Neurosurgery, University Hospital of Basel, Basel, Switzerland; 2 Division of Pediatric Neurosurgery, University Children’s Hospital of Basel, Basel, Switzerland; 3 Faculty of Medicine, University Hospital of Basel, Basel, Switzerland

**Keywords:** Growing skull fractures, Leptomeningeal cyst, Children, Surgery, Dural repair

## Abstract

**Background:**

Growing skull fractures (GSF) represent a rare complication following linear traumatic skull fractures with an underlying dural tear, mainly occurring during infancy and early childhood. GSF can cause encephalocele, hydrocephalus, or encephalomalacia, potentially leading to long-term neurological sequelae. Therefore, prompt diagnosis and early treatment is paramount.

**Methods:**

We outline our surgical reconstruction technique for managing GSF.

**Conclusion:**

GSF is effectively treated with a dural and bony reconstruction, using a fast-resorbable polymer mesh.

**Supplementary Information:**

The online version contains supplementary material available at 10.1007/s00701-025-06646-w.

## Introduction and relevant surgical anatomy

Growing skull fractures (GSF) were first described in 1816 by John Howship [1]. GSF occur in 0.05%−0.6% of linear skull fractures in infants and rarely in toddlers up to 3 years [6, 7, 10]. If left untreated, GSF can lead to encephaloceles, possibly causing serious neurological deficits [10]. This highlights the necessity of an accurate diagnosis and early treatment.

A linear skull fracture with an underlying dural tear, allowing the arachnoid membrane or brain tissue to entrap within the fracture margin, represents the pathophysiologic basis for GSF development. The pulsatile brain tissue within the fracture leads to an enlargement of the fracture by preventing osteoblasts from migrating to the fracture site [8, 9]. Additionally, the continuous pressure of herniated brain tissue contributes to resorption of the adjacent bone, leading to a fracture line progression [8, 9]. Hence, infants and children up to 3 years with a linear skull fracture should have a clinical follow-up within 6–12 weeks to rule out GSF [2].

## Description of the technique

The positioning is individual, given the location of the fracture. We either use a padded horseshoe or Doro Pediatric Skull Clamp (Doro® BlackForest Medical, Freiburg, Germany), maximizing access to the fracture site. Moreover, all pressure points should be padded sufficiently to prevent pressure sores.

**Fig. 1:**
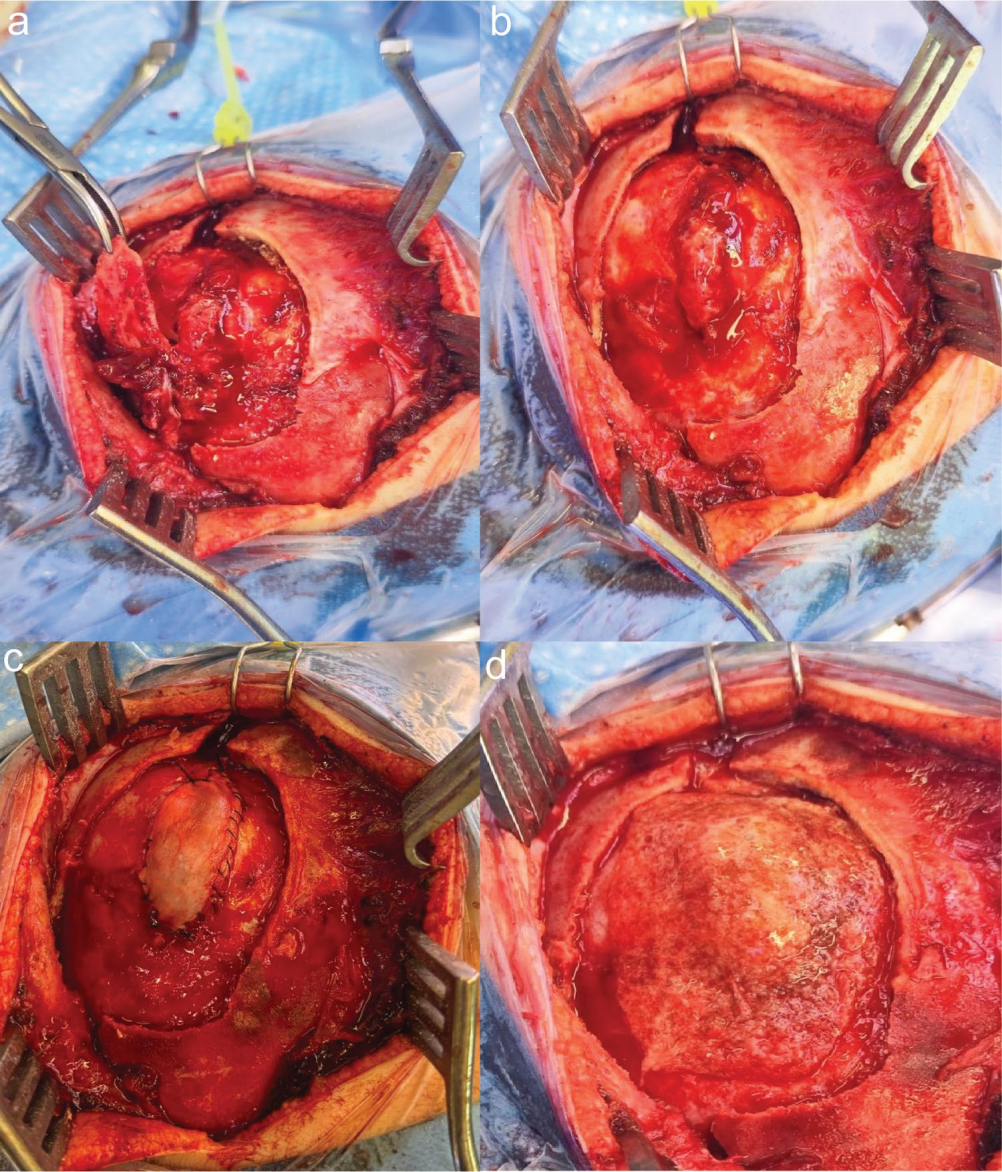
**a** Surgical site prior to craniotomy, but after loose bone fragments were removed. **b** Surgical site after the craniotomy. The craniotomy was performed to expose the whole dural defect including the encephalocele. Usually, one should aim at leaving at least a one-centimeter margin around the encephalocele. **c** Surgical site after resection of scarred encephalocele, correct replacement of the brain tissue, and dural closure with suturable duraplasty. The duraplasty should optimally be prepared to match the defect exactly and allow a proper placement and fixation, preferably in a sublay technique. **d** Surgical site after strengthening of the duraplasty using a layer of TachoSil (Takeda Pharmaceutical Co.)

The incision is tailored to the individual patient’s fracture location. The dissection should be performed carefully as the fracture is dehiscent, and potentially healthy brain tissue could be directly underneath the skin. The GSF is exposed in its entirety (Fig. 1a), and as the dural defect is frequently larger than the bony defect, the craniotomy should be fashioned with a margin around the dural defect, and if present, around the encephalocele (Fig. 1b). Whenever feasible, a single large bone flap is elevated en bloc, rather than utilizing a piecemeal bone removal technique.

After elevating the bone flap, the dural defect is fully exposed, allowing for a watertight dural repair. The use of a microscope is helpful at this stage to ensure minimal manipulation of herniated brain tissue (Video 1).

The crucial step of the procedure is achieving a watertight dural closure to promote optimal bone healing. Given that direct dural repair is usually not achievable due to the enlarging dural tear, a duraplasty might be required. We use a suturable duraplasty (Tutopatch, RTI Biologics; or Neuropatch, B. Braun), which is cropped to the desired shape and tightly attached in a sublay technique (Fig. 1c). To strengthen the dural reconstruction, a layer of TachoSil (Takeda Pharmaceutical Co.) and fibrin glue can be added (Fig. 1d**)**. The dural closure is considered watertight if no obvious cerebrospinal fluid (CSF) leakage is noted.

**Fig. 2:**
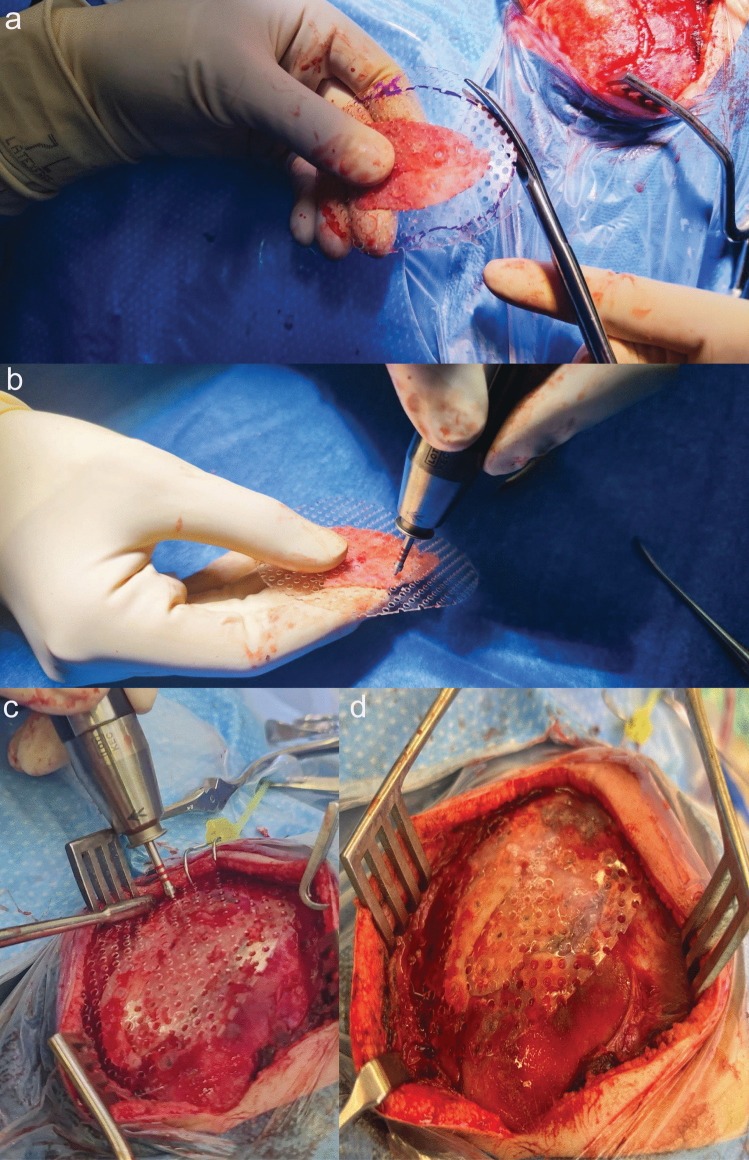
**a** The fast-resorbing polymer mesh is cut to fit the form of the bony defect. **b** The bone flap, including the bone fragments, are fixed and attached to the prepared mesh plate and fixed with resorbable screws.** c** The construct of the mesh plate and, if present, bone fragments, are attached to the bony defect. Fixation is performed with resorbable screws.** d** Surgical site after fixation of the bone fragments to the defect

Depending on the bony defect, the fragments/bone flap, are fixed using resorbable screws or a fast-resorbing polymer mesh plate (DePuy Synthes). The fast-resorbing polymer mesh plate is cut to match and overly the shape of the bone defect (Fig. 2a). The bone flap and/or the bone fragments are then attached to the polymer mesh plate (Fig. 2b), which in turn is fixed as a whole piece to the bone edges using resorbable screws (Fig. 2c and d). Ideally, the bone should cover the site of dural repair, consequently providing additional local pressure to prevent leaks. The skin is closed in layers with subcutaneous and cutaneous resorbable sutures.

The whole procedure and operating technique are visualized in Video 1.

## Indications

The existing literature agrees that a surgical intervention is necessary to treat GSF and avoid its progression [3, 5–10]. Hence, the indication is solely based on a reliable diagnosis of GSF.

## Diagnosis

GSF usually develops over 6–12 weeks after the initial trauma, leading to a delayed diagnosis when the skull defect has already extended. A GSF can be diagnosed during clinical examination by palpating a soft defect in the skull [2]. After clinical diagnosis, imaging such as ultrasonography (US), computer tomography (CT), or magnetic resonance imaging (MRI) is performed to confirm the diagnosis [4, 10]. US or B-mode echography through the bony defect can be used via the fracture line and has been reported to significantly contribute to the early detection of dural tears and, if present, herniated brain parenchyma [2], while CT and MRI can aid in surgical planning.

Therefore, a clinical follow-up of every simple skull fracture is recommended 6–12 weeks after the initial trauma in infants and children < 3 years. Dislocated fractures should be worked up with an MRI scan after the initial trauma to detect any dural tear, which could be missed in US or CT imaging [4].

## Limitations to surgical treatment

A dural repair is essential to avoid progressive enlargement of the fracture. However, dural repair can be challenging, particularly in large defects. Moreover, as direct head traumas often come with soft tissue injuries, some of these children could have a higher risk of inadequate wound healing [2, 4].

Of note, despite an early and adequately performed surgery, some infants and children continue to suffer from seizures, developmental delays and focal neurological deficits [5, 10].

## Preoperative management

An accurate and timely diagnosis is required to ensure early treatment. Hence, a follow-up of infants and children < 3 years of age with a diagnosed skull fracture, should be performed. In children presenting with a dislocated fracture, an MRI is recommended after trauma to assess for dural tear, to avoid GSF and to offer primary surgery to close the dura [4]. Once a GSF is diagnosed, prompt surgical repair should be performed to prevent further complications [2].

## Surgical management

During surgery, one should avoid aggressive dissection due to the underlying healthy brain tissue. Notably, the dural defect is often greater than the bony defect, and an adequate exposure is required for a successful closure. Meticulous dural repair is essential, with the bony reconstruction positioned primarily over the dural defect to exert local pressure and support healing. Use resorbable plates not to hinder skull growth of create deformities of the skull.

## Specific information for the patient

GSF are a rare complication of linear skull fractures, mainly occurring during infancy. An accurate, early diagnosis and prompt treatment are crucial to prevent secondary damage to the brain tissue, which can be associated with neurological sequelae. Generally, GSF are treated with surgical intervention, which is performed under general anesthesia. With timely surgery, the prognosis is favorable. It is paramount that parents should be informed about the risks of surgery, such as infection, CSF leakage, bone graft resorption, fracture recurrence, and persistent neurological symptoms in rare cases.

## 10 Key points summary

1. GSF are a rare complication of linear skull fractures, with more than 50% of cases occurring in children under the age of 12 months, and 90% occurring under the age of 3 years.
2. The patho-anatomical prerequisite for GSF development is a dural tear underneath the linear skull fracture.
3. Unlike typical fractures without a concomitant dural defect, GSF progressively enlarge due to the herniated pulsatile brain tissue, leading to expansion of the bony defect, which can lead to significant complications such as encephalocele, hydrocephalus, encephalomalacia, and irreversible neurological deficits.
4. GSF should be suspected if a history of traumatic brain injury with a skull fracture exists, particularly in children less than 3 years of age and if clinical symptoms such as neurological deficits related to fracture localization, epileptic seizures, and pulsatile scalp swelling are present.
5. Brain imaging (MRI, CT, US) is routinely used for the diagnosis of skull fractures and help to identify the extent of the fracture as well as the associated brain injury.
6. The dural defect can be larger than the bony defect, and the bone flap size should be planned and preformed accordingly.
7. Dissection should be performed carefully, ideally under microscopic view, to avoid injury to possible healthy brain tissue.
8. The crucial step of the surgical treatment of GSF is to achieve a watertight dural closure to promote optimal bone healing.
9. Cranioplasty can be performed using autologous bone fragments/bone flap, which are then assembled and attached to a polymer mesh plate.
10. As skull growth is still expected in this patient population, resorbable fixation systems should be preferred.

## Supplementary Information

Below is the link to the electronic supplementary material.

Supplementary Material 1 (MP4 178 MB)

## Data Availability

No datasets were generated or analysed during the current study.
